# Why the increase in under five mortality in Uganda from 1995 to 2000? A retrospective analysis

**DOI:** 10.1186/1471-2458-11-725

**Published:** 2011-09-25

**Authors:** Fred Nuwaha, Juliet Babirye, Natal Ayiga

**Affiliations:** 1 School of Public Health, Makerere University, P.O. Box 7072 Kampala Uganda; 2 Institue of Statistics and Applied Economics, Makerere University, P.O. Box 7072 Kampala, Uganda

## Abstract

**Background:**

From 1995-2000 the under five mortality rate in Uganda increased from 147.3 to 151.5 deaths per 1000 live births and reasons for the increase were not clear. This study was undertaken to understand factors influencing the increase in under five mortality rate during 1995-2000 in Uganda with a view of suggesting remedial actions.

**Methods:**

We performed a comparative retrospective analysis of data derived from the 1995 and the 2000 Uganda demographic and health surveys. We correlated the change of under five mortality rate in Uganda desegregated by region (central, eastern, north and western) with change in major known determinants of under five mortality such social economic circumstances, maternal factors, access to health services, and level of nutrition.

**Results:**

The increase in under five mortality rate only happened in western Uganda with the other 3 regions of Uganda (eastern, northern and central) showing a decrease. The changes in U5MR could not be explained by changes in poverty, maternal conditions, level of nutrition, or in access to health and other social services and in the prevalence of HIV among women attending for ante-natal care. All these factors did not reach statistical significance (P > 0.05) using Pearson's correlation coefficient.

**Conclusion:**

In order to explain these findings, there is need to find something that happened in western Uganda (but not other parts of the country) during the period 1995-2000 and has the potential to change the under five mortality by a big margin. We hypothesize that the increase in under five mortality could be explained by the severe malaria epidemic that occurred in western Uganda (but not other regions) in 1997/98.

## Background

The chance of dying before the fifth birth day (during childhood) otherwise known as the under-five mortality rate (U5MR) or childhood mortality rate, is a composite indicator measuring human as well as economic progress [1]. The U5MR measures an end result of the development process from multiple inputs such as income, income distribution, food availability in the family, the availability of clean water and safe sanitation, use of maternal and child health services (including prenatal care), level of immunization, and the overall safety of the child's environment [2]. Moreover, the U5MR is less susceptible than, say, per capita Gross Domestic Product (GDP) to the fallacy of the average [3]. This is because the natural scale does not allow the children of the rich to be one thousand times as likely to survive, even if the man-made scale does permit them to have one thousand times as much income. In other words, it is much more difficult for a wealthy minority to affect a nation's U5MR, and the indicator therefore presents a more accurate, if far from perfect, picture of the health status of the majority of children (and of society as a whole) [1,3].

The United Nations and its agencies use U5MR as measure of wellbeing and consequently decreasing the U5MR is included in the millennium development goals (MDGs) [4]. According to the MDG number four, Uganda is expected to reduce U5MR by two thirds from 195 deaths per 1000 (in 1990) to 65 deaths per 1000 in 2015. In order to achieve this goal, the annual average reduction rate (AARR) of U5MR should be more than 4.4% [5]. Recent analyses showed that the AARR of 4.4% has never been reached in Uganda [6]. Besides, according to the Uganda Demographic Health Surveys (UDHS) of 1995 and 2000, the U5MR increased from 147.3 deaths per 1000 live births to 151.5 deaths per 1000 live births [7,8]. The reasons for this increase in U5MR are not clearly known and there has been a lot of speculation both internationally and locally regarding possible causes [9]. Moreover, the increase appeared paradoxical since the major determinants of U5MR such as GDP per capita, prevalence of HIV among pregnant women, and the quality and quantity health services and other social services were favourable during the same time [10]. There is thus need to the understand factors that could have been responsible for the increase in U5MR during the period 1995-2000 in the country. It has been stressed that understanding of factors influencing changes in U5MR at country level rather than in geopolitical areas is a pre-requisite for designing effective intervention methods aimed at decreasing childhood mortalities [11]. This article reports on factors that most likely influenced the increase in the under five mortality rate in Uganda for the period 1995-2000.

## Methods

### Setting

Uganda is a low-income country by all indicators with current projected population of 32 million people (from the 2002 national census at annual population growth rate of 3.4%). The social-economic development has been characterised by political upheavals since 1970s, resulting into two major wars during 1978/79 and during 1981-1986. During this period there was a reversal in social-economic development with shrinkage of gross domestic product per capita (GDP) in terms of purchasing power parity (PPP) from US$ 615 in 1969 to 443 in 1980 [11]. This trend appears to have been reversed in 1986. Since then the economy has been growing at an annual rate of over 5% far ahead of population increase estimated at about 3% during the same period [9]. The economy of the country is predominantly dependant on agriculture for more than 80% of the employment. Land ownership is almost universal in rural areas where more than 87% of the population live. As a result the gini coefficient (which is a measure of income distribution) in the country is favourable and during the 1995-2000 period varied from 0.35 to 0.38 [12].

**Figure 1:**
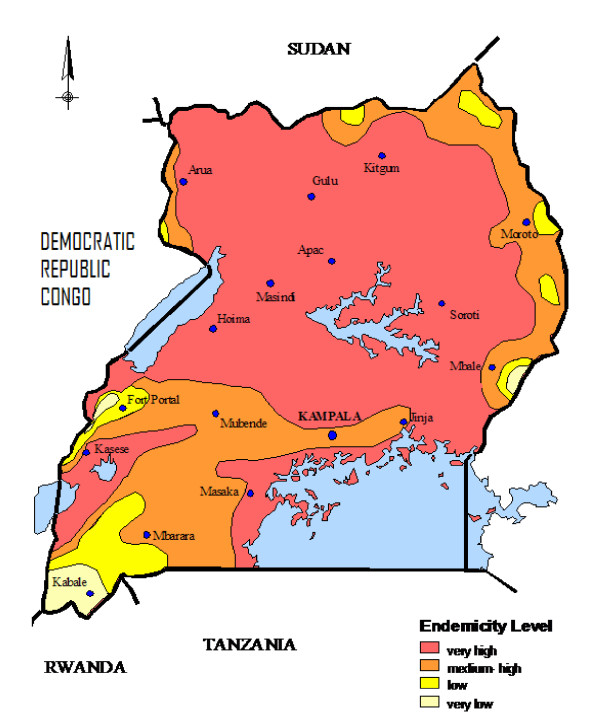
**Map of Uganda showing malaria endemicity**.

The major factors influencing childhood mortalities in the country include maternal conditions (such as education, parity, age) birth order, nutritional status of the child, place of residence (rural or urban), HIV prevalence rates among pregnant women, malaria endemicity, wealth of households and place of delivery for newborns [7,8]. The fundamental direct causes of childhood mortalities include: peri-natal conditions (such as pre-maturity, low birth weights and level of supervision during child birth), malaria, diarrhoea, pneumonia, HIV/AIDS, malnutrition and measles. These 7 conditions are responsible for more than 90% of the total childhood mortalities [10]. The HIV infection rate in the country was highest in the early 1990 but has since started declining and it now stands at less than 7% of the adult population [13]. Uganda broadly has two types of malaria transmission whereby about 90% of the country lies in stable malaria transmission (predominantly in the eastern, northern and central parts of the country) and about 10% (that is predominantly in the western region of the country) is characterised by unstable malaria transmission and prone to epidemics (see figure 1) [14]. Although the land area of unstable malaria transmission is only about 10%, the population density of malaria free areas and low transmission areas in western Uganda is very high. As such about one fifth of the Ugandan population on the whole live in either malaria free or low transmission areas in western Uganda [14,15]. Malaria in low transmission areas of western Uganda is characterised by epidemics. The worst malaria epidemic characterised by very high childhood mortalities occurred in western Uganda in 1997/1998 and was greatly influenced by the *el-Niño *weather phenomenon [16-18].

### Data sources and analyses

Data were abstracted from the 1995 and 2000 Uganda demographic and health surveys [7,8]. The UDHS of 1995 and 2000 were designed to have adequate sample sizes (7070 and 7246 women aged 15-49 respectively) proportionate to population of the regions to allow for estimation of childhood mortality indices by the four regions of Uganda. In both surveys, data were collected on characteristics of household members, socio-economic status of respondents and of households, fertility regulation, determinants of fertility, fertility preferences, reproductive health and child care, nutritional status of children, morbidity in previous two weeks, adult mortality, and HIV/AIDS. Both surveys used the same methodology and collected data on retrospective birth histories which provided direct estimates of childhood mortality [19]. The analysis of 1995 and 2000 UDHS data included disaggregation of under-five mortality data by age (e.g. within first month of life (neonatal mortality rate-NMR), between second month of life and before 12 months of life (post neonatal mortality-PNMR), during the first year of life (infant mortality rate-IMR), during years 1-4 of life (child mortality rate-CMR) and during the first five years of life (under five mortality rate-U5MR). The mortality data was also disaggregated by the four regions of Uganda (Central, Eastern, Northern and Western).

Data were also abstracted on possible covariates of childhood mortality such as female education, proportion of population living below the poverty line, proportion of mothers delivering under medical supervision, percentage of mothers with high risk pregnancies, rates of usage of modern family planning, rates of measles immunization, HIV infection among antenatal mothers, childhood malnutrition, and incidence of fever, diarrhoea and cough/rapid breathing within the previous two weeks, and with the occurrence or non occurrence of the malaria epidemic in 1997/1998. Economic indicators such as proportion of population living below the poverty line were derived from Uganda national and household surveys (UNHS) of 1992 and of 1999 [12,20]. These surveys had sample sizes between 5000-10,000 households and were designed to capture regional differences particularly among proportions of the population living below the poverty line. The rates of HIV infection among women attending antenatal care (ANC) were derived from surveillance data which has been collected from a representative sample of women in various health units in the country since 1989 [21]. Other measures were obtained from the UDHS of 1995 and 2000 [7,8].

### Statistical Analysis

Ninety five percent confidence intervals (95% CI) of mortality rates were used to gauge whether there were significant changes in mortality indices between 1995 UDHS data and that of 2000. We further analysed association between changes of various determinants of U5MR by region with changes in U5MR between 1995 and 2000 based on the method of concomitant variation [22]. This method is used to ascertain the relationship between two variables to establish causality based on the fact that if two phenomena vary up and down simultaneously, one is causing the other or there is a third factor causing both of them [23]. The deviations of an oscillatory variable with respect to its rate of change, measures both the size and the direction of its change over time. Therefore if two variables oscillate simultaneously, because one causes the other or a third factor causes both, their rates of change will be highly correlated. In order to apply the method of concomitant variation, the variables were transformed into rates of change (i.e. the ratio X2000-X1995/X1995)*100 expressing the relative change from the year 1995 to the year 2000 into a percentage. Correlations between the transformed variables and the change in U5MR by region were then computed. Significance of change in determinants with change in U5MR were tested using Pearson's correlation coefficient (r) using a two tailed test at 5% level of significance.

### Ethical considerations

The Uganda National Council for Science and Technology (UNCST) and the Makerere University Institute of Public Health (MUIPH) higher degrees and ethics committee independently approved the study. Prior to data collection permission was sought from the relevant Uganda government authorities.

## Results

**Figure 2:**
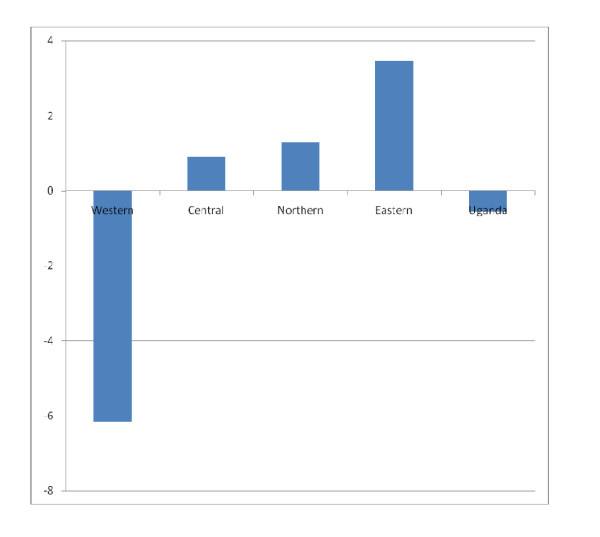
**Annual average reduction rate (AARR) of U5MR in Uganda by region 1995-2000**.

Table 1 shows the childhood mortality rates in Uganda disaggregated by age of the child and by region for the period 1995-2000. It is interesting to note that only the western region showed an increase in U5MR whereas other regions of the country showed a decrease (figure 2). Furthermore, only in western Uganda did the change in U5MR reach statistical significance (i.e. the 95% confidence interval for the years 1995 and 2000 do not overlap). In western region most of the observed increase in U5MR is contributed by an increase in neonatal mortality and by increase in child mortality. Figure 2 shows that the AARR was most remarkable in terms of magnitude and direction (being negative) in the western region compared to other regions of the country.

**Table 1:** Changes in Childhood mortalities in Uganda 1995-2000 by country and region

	1995			2000		
	Rate (Per 1000)	95% CI	Weighed Sample	Rate (Per 1000)	95% CI	Weighed Sample
**Uganda**						

NNMR	27.0	22.1-31.9	7681	33.2	27.8-38.5	7834
PNMR	54.3	47.0-61.6	7717	55.2	47.9-62.6	7853
IMR	81.3	71.1-90.4	7719	88.4	78.8-98.0	7854
CMR	71.9	63.6-80.2	7834	69.2	60.0-78.4	8007
U5MR	147.3	135.1-159.5	7875	151.5	138.2-164.8	8028

**Central**						

NNMR	29.6	21.9-37.2	3579	29.8	23.4-36.1	4039
PNMR	47.0	37.6-58.4	3585	42.2	33.2-51.2	4042
IMR	76.6	63.9-89.3	3586	71.9	60.0-84.0	4044
CMR	70.1	57.8-82.4	3612	68.1	52.8-83.4	4082
U5MR	141.3	122.2-160.5	3621	135.1	113.6-156.6	4088

**Eastern**						

NNMR	38.4	31.1-45.7	3615	29.5	21.6-37.4	4201
PNMR	59.7	50.0-69.5	3623	59.8	48.2-71.4	4204
IMR	98.1	84.9-111.3	3623	89.3	74.7-104.0	4204
CMR	86.0	71.6-100.4	3665	63.7	52.3-75.1	4231
U5MR	175.7	156.5-194.6	3672	147.3	131.5-163.1	4234

**Northern**						

NNMR	33.6	23.8-43.4	2653	42.2	31.6-52.7	2413
PNMR	65.8	49.2-82.3	2655	63.7	49.3-78.1	2421
IMR	99.3	78.7-120.0	2655	105.9	89.7-122.0	2421
CMR	100.6	73.1-128.1	2693	80.6	63.3-97.9	2439
U5MR	190.0	162.6-217.3	2965	178.0	155.4-200.5	2447

**Western**						

NNMR	26.8	20.7-32.9	4029	41.5	33.8-49.2	3586
PNMR	48.3	37.2-59.5	4030	56.3	44.3-68.3	7853
IMR	75.1	61.3-89.0	4032	97.8	82.3-113.4	3597
CMR	60.1	47.4-72.8	4057	87.0	69.5-104.5	3638
U5MR	130.7	110-150.1	4061	176.3	152.3-200.3	3638

The possible determinants of U5MR for the year 1995 and 2000 are shown in table 2. Data from the UDHS of 1995 and 2000 [7,8] and table 2 show that the western region has a significantly lower proportion of women who deliver under medical supervision for both 1995 (24.1%; 95% CI 18.9-29.2 compared to 37.8%; 95% CI 35.0-40.6 for Uganda) and 2000 (23.1%; 95% CI 19.7-26.5) compared to the national average (38.2%; 95% CI 35.4-41.0). Other indicators that are also unfavourable in western Uganda compared to the national average include: the much lower level of pregnant women receiving anti-malarial chemoprophylaxis during pregnancy for the year 2000 (23.0% for western Uganda versus the national average of 33.8%) and the percentage of household with bed nets (5.5% for western Uganda versus national average of 12.8%). Moreover, the bed nets in western region were more likely to be owned for less than 13 months compared to national average of 28 months. It was not possible to measure change in households which own bed nets as the 1995 UDHS did not collect this data [7].

**Table 2:** Correlations of changes of possible determinants with changes in Under-five mortality in Uganda by region 1995-2000

Determinant/Region	Rate or proportion in 1995	Rate or proportion in 2000	Effect of change on U5MR	Pearson correlation coefficient (r) and (p-value)
Percent of population living below poverty line				

Central	46	28	Decrease	
Eastern	63	58	Decrease	
Northern	73.4	57.4	Decrease	
Western	49.2	34.2	Decrease	-0.51 (0.49)
Uganda	56	38	Decrease	

Percent of urban population				

Central	35.1	36	Decrease	
Eastern	9.3	10.3	Decrease	
Northern	7.0	6.7	Increase	
Western	5.4	4.7	Increase	-0.88 (0.12)
Uganda	14.9	16.7	Decrease	

Percent of women who have never attended school				

Central	20.5	19.4	Decrease	
Eastern	34.9	23.3	Decrease	
Northern	52.0	39.4	Decrease	
Western	39.2	30.9	Decrease	0.22 (0.78)
Uganda	35.6	26.5	Decrease	

Percent of children malnourished (< 2SD weight for height)				

Central	3.5	3.6	Increase	
Eastern	6.6	4.3	Decrease	
Northern	4.2	3.8	Decrease	
Western	4.1	4.3	Increase	0.71 (0.29)
Uganda	5.3	4.0	Decrease	

Percent of children > 9 months who received measles immunisation				

Central	65.8	50.9	Increase	
Eastern	48	53.1	Decrease	
Northern	51.5	57.9	Decrease	
Western	72	66.9	Increase	-0.38 (0.62)
Uganda	59.6	56.8	Decrease	

Percent of pregnant women who delivered under medical supervision				

Central	59.9	58.8	Increase	
Eastern	41.3	40.2	Increase	
Northern	22.6	26.8	Decrease	
Western	24.1	23.1	Increase	-0.33 (0.67)
Uganda	37.8	38.2	Decrease	

Percent of children with cough and rapid breathing in previous 2 weeks				

Central	21.4	19.4	Decrease	
Eastern	23.0	23.3	Increase	
Northern	30.8	23.1	Decrease	
Western	33.7	24.6	Decrease	-0.70 (0.30)
Uganda	27.1	22.5	Decrease	

Percent of children with reported fever in previous 2 weeks				

Central	39.3	37.9	Decrease	
Eastern	58.8	54.1	Decrease	
Northern	58.2	50.4	Decrease	
Western	32.6	33.8	Increase	0.90 (0.10)
Uganda	46.3	43.9	Decrease	

Percent of children with diarrhoea in previous 2 weeks				

Central	16.3	14.5	Decrease	
Eastern	26.2	23.3	Decrease	
Northern	34.3	26.7	Decrease	
Western	20.0	16 0	Decrease	-0.56 (0.44)
Uganda	23.5	19.6	Decrease	

Median number of months since previous birth for mothers				

Central	28.7	28.7	No change	
Eastern	28.6	28.3	Decrease	
Northern	31.8	31.8	No change	
Western	29.3	29.4	Increase	0.71 (0.29)
Uganda	29.3	29.2	Decrease	

Adolescent pregnancy (percent of mothers who began child bearing at 15-19 years)				

Central	42.7	31.2	Decrease	
Eastern	51.1	36.5	Decrease	
Northern	41.4	34.3	Decrease	
Western	37.2	24.3	Decrease	-0.63 (0.37)
Uganda	42.9	31.4	Decrease	

Percent of women who received anti-malarial during Antenatal care (ANC)				

Central	34.9	39.7	Decrease	
Eastern	39.4	39.2	Decrease	
Northern	31.4	30.4	Decrease	
Western	23.4	23.0	Increase	-0.17 (0.83)
Uganda	34.6	33.8	Decrease	

Percent of pregnant women attending ANC who are HIV positive				

Central	20.2	10.7	Decrease	
Eastern	12.5	4.7	Decrease	
Northern	16.3	11.3	Decrease	
Western	16.6	10.0	Decrease	0.42 (0.58)
Uganda	15.6	7.5	Decrease	

Correlations are for changes in four regions (Central, Eastern, Northern, and Western) but not Uganda.

As shown in table 2 correlation coefficients for all possible determinants of U5MR for the period 1995 and for 2000 were not statistically significant (P > 0.05). It is also interesting to note that most of the determinants were favourable for the year 2000 compared to 1995 and the expected effect on U5MR would have been a reduction not an increase!

## Discussion

These data show that the period 1995-2000 was characterised by an increase in U5MR in Uganda from 147.3 to 151.5 deaths per 1000 live births. Disaggregation of the data shows the increase of U5MR in the country was only contributed by the western region with other regions of the country showing a decline in U5MR. Furthermore, this analysis demonstrated that the increase in U5MR in the western region could not be explained by the changes in the major determinants of childhood mortalities such as changes in poverty levels, quality and quantity of health and other social services.

In order to explain the observations related to changes in U5MR in Uganda for the period 1995-2000, there is need to find something that happened only in western Uganda during the period 1995-2000 and has the potential to drastically change the U5MR by a big margin. It is hypothesized that this increase in U5MR in western Uganda could be attributable to the severe malaria epidemic that happened in western Uganda in 1997/1998. This was the worst malaria epidemic happening in Uganda and it was characterised by *el-Niño *weather phenomenon with almost twice the level of rainfall [16-18].

Several attributes makes the malaria epidemic in western Uganda a likely explanation for the observed trends in childhood mortality. First, there is temporal sequence [24]. The increase in childhood mortality was preceded by the malaria epidemic which followed the *el-Niño *weather phenomenon [16-18]. Second the malaria epidemic happened in western Uganda only and this is the same region that experienced the increase in childhood mortalities. Third the assertion linking malaria as a major determinant of U5MR is consistent with other studies that demonstrated an increase of childhood mortality with occurrence of malaria epidemics in various parts of Africa [25,26] or that show relative low childhood mortalities in malaria free areas [27]. For example, according to the 2002 census in Tanzania the U5MR for relatively malaria free areas of Arusha and Kilimanjaro regions were 58 and 67 deaths per 1000 live births respectively compared to the U5MR for Tanzania (154 deaths per 1000 live births) and the U5MR for Dar-es-salaam (123 deaths per 1000 live births) [27]. Furthermore, data from intense malaria control strategies also show that control of malaria reduces the overall U5MR by up to 60% [28].

The explanation that malaria epidemics increase U5MR is biologically and theoretically plausible from several perspectives. First, malaria is more likely to affect children aged 0 to 4 years hence the corresponding negative consequences on childhood mortalities [29-31]. Although, malaria causes significant childhood mortality in areas of stable high transmission, the effect of malaria on childhood mortality under such conditions of transmission is stable and therefore not detectable by time trends. Moreover, under conditions of stable transmission children in the first 2 years of life are more susceptible to malaria whereas in unstable transmission areas the effect of malaria on mortality is felt throughout all the years of childhood [29]. Thus, the negative effect of malaria on childhood mortality for unstable malaria is more likely to be more than that of stable malaria. Second, in stable transmission areas malaria in pregnancy predominantly affects prime-gravida (that forms only about 15% of total mothers who become pregnant) whereas in unstable transmission areas malaria affects both prime and multi-para (100% of pregnant women) [32,33]. Therefore, the effect of malaria epidemics on childhood mortality by this factor is that many more children in unstable malaria areas are affected compared to children in stable malaria areas. Third, unstable malaria is more likely to lead to prematurity in low transmission areas whereas in high transmission areas malaria infection in pregnancy is more likely to lead to underweight [29,32,33]. Although, both underweight and prematurity increases the risk of children death, the risk of premature deaths is about twice that of underweight children [34]. Fourth, the effects of anti-malarial drug resistance on mortality are more likely to be felt among the non-immune as found in low transmission areas compared to the semi-immune that are more likely to be found in high transmission areas [35]. Indeed, at the time of the 1997/98 epidemic, the malaria treatment policy was still chloroquine whose level of resistance in Uganda particularly in the western region was very high [36]. Finally, another factor that leads credence to these assertions is that the western region of Uganda according to the 2000 UDHS [8] was the least prepared to prevent malaria or to mitigate its effects. For example the percent of household using bed nets was 5% in western Uganda compared to 13% for the rest of the country; the percent of pregnant women receiving malaria prophylaxis was 21% for western Uganda compared to 40% for the rest of the country and the percent of women delivering under medical supervision was 22% in the west compared to 37% in Uganda.

A limitation of this study was that information used in the analyses was collected retrospectively and therefore susceptible to recall bias especially on the age of death, where respondents could have forgotten events that happened in the past. We think that this bias was spread through all the regions of Uganda and therefore less likely to affect trends. Another limitation is that since most of the women in Uganda deliver at home and their children are not weighed at birth; it was thus not possible to analyze the trends of prematurity and low birth weight for adducing more evidence. However, data from neighbouring Tanzania suggest that during the 1997/1998 *El Niño *weather phenomenon there was an epidemic of low birth weights especially in low malaria transmission areas [37]. Besides, the 2000 UDHS demonstrated that the western region had a more unfavourable peri-natal mortality of 52.6 per 1000 pregnancies compared to the national average of 42.6 per 1000 pregnancies [8]. The peri-natal mortality is a measure of still births and deaths within the first week of life. Prematurity, low birth weight, malaria and home deliveries are major determinants of perinatal deaths [38].

Nevertheless our methods indicate the utility of disaggregated data using ecological evidence to understand events that would otherwise be unethical to study using randomization. Besides, these data are almost as good as a natural experiment where events were analyzed before and after the possible determinant occurred and in places where the possible determinant occurred and did not occur [39].

## Conclusion

Thus it is highly plausible that malaria was a major determinant of childhood mortality in Uganda during the period 1995-2000. The mechanisms through which malaria causes childhood deaths are both direct and indirect. Directly malaria increases childhood deaths through causing infection and severe disease among affected children. Indirectly malaria causes deaths by leading to low-birth weight and prematurity. Thus in order to speed the reduction in childhood mortality in Uganda, there is need to aggressively control malaria as through universal coverage with intermittent presumptive treatment in pregnancy (IPT), use of long lasting insecticide treated nets, residual household spraying and increasing access to prompt treatment [40]. Additionally, there is need to increase the proportion of children born under qualified medical supervision through improvement in peri-natal health care and in increasing the number of health personnel that can supervise deliveries [41].

## Competing interests

The authors declare that they have no competing interests.

## Authors' contributions

FN contributed to the study concept, and design, analysis of the data, writing and editing of the paper. JB contributed to study design, collection of data, analysis and in writing and editing of the paper. NA contributed in collection of data, analysis and in writing of the paper. All authors read and approved the final manuscript.

## Pre-publication history

The pre-publication history for this paper can be accessed here:

http://www.biomedcentral.com/1471-2458/11/725/prepub
